# Approaching Collectivity Collectively: A Multi-Disciplinary Account of Collective Action

**DOI:** 10.3389/fpsyg.2021.740664

**Published:** 2021-12-02

**Authors:** Gerhard Thonhauser, Martin Weichold

**Affiliations:** ^1^Department of Philosophy, Technical University of Darmstadt, Darmstadt, Germany; ^2^Institute of Philosophy, University of Regensburg, Regensburg, Germany

**Keywords:** collective action, collective intentionality, collective affordance, collective sense-making, ecological social psychology, practice theory, subjectification, collectification

## Abstract

There has been considerable progress in investigating collective actions in the last decades. However, the real progress is different from what many scholars take it to be. It lies in the fact that there is by now a wealth of different approaches from a variety of fields. Each approach has carved out fruitful mechanisms for explaining collective action, but is also faced with limitations. Given that situation, we submit that the next step in investigating collective action is to acknowledge the plurality of approaches and bring them into dialogue. With this aim in mind, the present article discusses the strengths and weaknesses of some of the to our mind most relevant approaches to collective action in current debates. We begin with the collective intentionality framework, the team reasoning approach, and social identity theory. Then, we move to ecological social psychology, participatory sense-making, and, through the lenses of those frameworks, dynamical systems theory. Finally, we discuss practice theory. Against this background, we provide a proposal for a synthesis of the successful explanatory mechanisms as they have been carved out by the different research programs. The suggestion is, roughly, to understand collective action as dynamical interaction of a self-organizing system with its environment, shaped by a process of collective sense-making.

## Introduction

There has been considerable progress in investigating collective actions in the last decades. However, the real progress is different from what many scholars take it to be. For, many scholars working on collective action study the phenomenon within the particular conceptual framework of their respective school of thought. These frameworks have advanced considerably in the last time. Yet each of the frameworks, we claim, also has limitations. The real progress in the investigation of collective action lies in the fact that there is by now a wealth of different approaches. Each approach has carved out fruitful mechanisms for explaining collective action, but is also faced with limitations. Given that situation, we submit that the next step in investigating collective action is to explicitly acknowledge the plurality of approaches, bring them into dialogue, and investigate which parts of them might be fruitfully combined. The present article aims at contributing toward that goal.

There are two ways in which the present article aims at achieving that goal. The first way is to provide an overview over seven to our mind highly relevant approaches to collective action. This is meant as a service to the reader, as we assume that part of the reason for why there is so little interaction between the different accounts is that not everyone is familiar with all of them. We acknowledge, of course, that there are also researchers who mindfully embrace only one of the approaches while they are fully aware of the existence of the other accounts. To those readers, it matters that we also discuss the strengths and weaknesses of each account – arguing that no account is perfect on its own. The price for investigating seven different accounts in one paper is that each discussion has to be briefer than it could otherwise have been. Yet we hold that this is still a price worth paying in light of the prospect of bringing those different accounts together for the first time.

The second of the two ways in which the present article aims to achieve its goal of bringing the different approaches into dialogue is to offer a first proposal of how the convincing core ideas of the different approaches can be integrated into a synthesized framework. We have no illusions here: There are crucial theoretical tensions between core assumptions of the different frameworks. However, we do (of course) not propose to synthesize all of the theoretical commitments of the seven different frameworks of collective action. We rather suggest that each approach has developed one (or two) helpful mechanism(s) for explaining collective action, and that these different mechanisms can be fruitfully synthesized, independently from the frameworks they are extracted from. Future work on further integration of the different frameworks can then take place against this background. In our view, collective actions are characterized by a variety of different, interconnected aspects, such as being structured by collective intentions, entailing dynamical sensorimotor interaction, or being embedded in social practices. Each of the different theoretical approaches highlights an important aspect or facet of the complex phenomenon of collective action, and provides a good explanation of the respective aspect. However, the proponents of the different approaches are often in danger of neglecting the aspects their approach is not focused on, or of even stipulatively excluding them from the realm of ‘proper collective action,’ or of providing mere *ad hoc* explanations. By contrast, one can gain better explanations if one combines the fruitful explanatory mechanisms and shows how they even gear into each other.

To be sure, when we use the term “explanatory mechanism,” this should not be confused or associated with “mechanistic explanations.” We do not aim for any mechanistic or reductionist account of collective action. Instead, we understand the term “explanatory mechanisms” as referring to sub-processes and aspects of the rich phenomenon of collective action, such that understanding the inner workings of a respective sub-process or aspect contributes to better understanding how collective actions work on the whole.

We start this article with discussing the prominent collective intentionality framework in the section called “Collective Intentionality,” highlighting its strengths, but also showing its limitations. In the section called “Team Reasoning,” we turn to the team reasoning approach, before presenting and analyzing social identity theory in the section called “Social Identity Theory.” From the section called “Ecological Social Psychology” onward, we turn to approaches that are radically opposed to the earlier, more ‘cognitivist’ approaches. In the section called “Ecological Social Psychology,” we investigate ecological social psychology, that is, ecological psychology’s treatment of the social. In the section called “Participatory Sense-Making,” we turn to enactivist cognitive science and to the participatory sense-making approach in particular. In the section called “Practice Theory,” we move from cognitive science to social theory, and in particular to practice theory. Finally, in the section called “A Synthesis” we provide our tentative suggestion of how the fruitful explanatory mechanisms uncarved by the different approaches from the different fields can be synthesized into an integrated framework for explaining collective action. We are aware that there are approaches to collective action which we do not consider in this paper, e.g., approaches from social neuroscience. But we insist that this paper makes a helpful start in bringing different accounts into contact, and that it will be clear from our synthesis where other approaches (e.g., from social neuroscience) would fit in.

Before we embark on the mentioned journey, we want to give an indication of how our proposal of synthesizing the different approaches will look like. To our mind, a successful explanation of collective action has to account for the fact that collective actions are performed by collectives. In other words, *collective action is the action of a collective*. However, appreciating this at first sight rather natural thought is hard for many thinkers. For, they assume that the only true agents that exist are individual agents. An alleged collective agent is then thought to be a mysterious entity, unless it can be reduced to the actions of individuals. By contrast, we conject that this way of thinking about individual agency has a contingent historical background (Siedentop, 2015) and is deeply problematic. To break free from such an individualism about agency, one should turn to the theory of dynamical self-organization. According to this way of thinking, a single human being is essentially a self-organizing system. Likewise, collectives are self-organizing systems that emerge whenever appropriately prepared human beings are related to each other and interact with each other in the right way. *Collectives* are neither static entities nor mere illusions, but *emergent systems that are characterized by a process of constant self-organization*. The behavior of a collective regulates the behavior of the involved human beings and is at the same time regulated by their behavior (this is the first aspect of our integrated framework, *dynamic self-organization*). For example, what a football team does is regulated by what its members do, but what its members do is regulated by the fact that they are members of a team in action. Importantly, the collective actions performed by such a collective are not only guided by its inner organization, but also by what the environment affords to the collective (*collective affordances*). More precisely, the action is guided by what the collective *perceives* the environment to afford. But how the collective perceives, or interprets, its environment is itself a process of collective sense-making (*collective sense-making*). Importantly, the collective as a whole and each of its members needs to make sense of the world from the perspective of the collective (*collective perspective*). But how is it possible that the members of a collective get started to make sense of the world from the perspective of a collective, at a time when the collective has not yet emerged due to dynamical self-organization? Here, it is crucial that the members already have social identities of being part of a collective, and that those social identities can become salient under the right circumstances (*embodied social identity*). Yet, why is it that certain human beings have certain social identities? To explain this, one should refer to the histories of the respective human beings. Through skill acquisition, habit formation, subjection to norms, mutual recognition, and other means humans are formed into beings that are ready to play their part in a collective (*subjectification*). However, what holds for a single human being as a self-organizing system also holds for a collective. How a collective behaves is also shaped by the history of the collective’s interaction with its environment, which modifies the collective sense-making dynamics and thereby the collective’s disposition for future interactions (*collectification*). Finally, on a macro level, many collective actions are structured by the goals and intentions of the collective and of its members (*collective goals and intentions*). To our mind, each collective action is characterized by those aspects, although in varying degrees. In some collective action, such as the actions of a sports team, a sensorimotor interaction with collective affordances is more important. In other collective actions, subjectification is more important, and in still other actions, collective intentions are most crucial. However, even a sports team needs collective intentions, and its members also need to be appropriately subjectified. Each collective actions thus needs all or nearly all of the explanatory mechanisms in order to be explained in all of its aspects. Even people who pursue a collective goal by each doing her own part in her own way (e.g., try to collectively find a missing wallet in the park by each searching in her own way; Searle, 1990), need at least a minimal way of dynamical self-organization, e.g., while negotiating the collective goal. The synthesis which we will develop at the end of the paper shows how one can conceptualize the explanatory mechanisms in such a way that they gear into each other. Still, this is not meant as an analysis of the *concept* “collective action” by means of providing necessary and sufficient conditions, but rather as an explanation of the *phenomenon* of collective action (see Weichold and Rucińska, 2021 for this distinction). Yet to begin with, we will first provide our overview and discussion of the seven different approaches to collective action.

## Collective Intentionality

A good starting point for our endeavor is the debate about collective intentionality, as this debate is particularly well-known, especially amongst analytic philosophers (for an introductory overview see Schweikard and Schmid, 2013). The debate is so broad ranging, both regarding the subject areas covered and the proposed theories and concepts, that it is impossible to do justice to the entire field here. Instead, we focus on the central feature of what we suggest calling the *collective intentionality framework* for conceptualizing collective action: the commitment to analyzing collective action by means of collective intentions. To review this core commitment, let us briefly go back to the origins of the current debate. It began with the question: How can we distinguish between a mere aggregation of individual actions and genuine collective action (Searle, 1990)? To take Gilbert’s (1990) example, what is the difference between each of us taking a walk for herself and the case of us taking a walk together? Now, the core intuition stipulating the debate is that we cannot account for the difference solely in terms of observable behavior, because there might be cases in which an aggregation of individual actions looks exactly like a collective action to the observer. For example, two unrelated hikers might coincidentally hike the same trail at the same speed right behind each other, just like a hiking group of two would. Based on this intuition, everyone working within the collective intentionality framework agrees that we need to account for the difference between parallel individual actions and collective action in terms of collective intentionality.

The big disagreement in the debate is about the nature of such intentionality. The initial debate was centered around the question what exactly makes a collective intention collective: Does the collectivity reside in the content, mode, or subject of the intention (Schmid, 2018)? Another disagreement concerns the role of normativity in collective action, with normativists like Gilbert claiming that all cases of collective action involve normative relations, while non-normativists like Bratman argue that there are at least some cases of collective action that do not involve normative relations (Gomez-Lavin and Rachar, 2018). Another point of debate is the underlying understanding of intention. While most early contributors to the debate subscribed to an understanding of intention as a psychological attitude (Bratman, 1987), some later contributors follow Anscombe (1963) in understanding intentions not as mental states, but as fundamentally related to the being or doing of an action (Stoutland, 1997; Thompson, 2011). Despite these differences, all contributors agree that collective intentions are the threshold for collective action. In other words, they consider it necessary for collective action that the partaking individuals have collective intentions.

Of course, scholars subscribing to the collective intentionality framework will most likely agree that an explanation of action in terms of intentions needs to be supplemented by additional explanatory mechanisms, for example by theories that account for processes on the sensorimotor level. The collective intentionality framework only entails that collective intentions are necessary for collective action, not that they are sufficient. Thus, this framework is open to integrating other approaches to collective action that account for additional conditions of (collective) action. At the same time, we are afraid that there are two implicit commitments pervasive within the collective intentionality debate that are holding back attempts to combine research on collective intentions with successful explanatory mechanisms from other fields.

First, we worry that traditional approaches to collective intentionality are too cognitivist to fully appreciate all aspects of collective action. The debate about collective intentionality began in the 1980s and 1990s and the first generation of scholars initiating the debate (Tuomela and Miller, 1988; Gilbert, 1990; Searle, 1990; Bratman, 1992) has been fully committed to the dominant theory of mind at the time – *cognitivism*. Over the last three decades, however, there is growing evidence that behavior is not always guided by contentful intentions, nor by the processing of information or content in general (Varela et al., 1991; Haugeland, 1998; Thompson, 2007; Hutto and Myin, 2013, 2017). By contrast, research has shown that human behavior regularly unfolds without the guidance of conscious intentions (Bargh and Chartrand, 1999; Dreyfus, 2005; Rietveld, 2008; Kahneman, 2011; Di Nucci, 2013; Romdenh-Romluc, 2013; Weichold, 2015; Davis, 2016). Indeed, it has been argued that even collective action is often not guided by collective intentions (Baier, 1997). Contrasting those findings with the above mentioned classics of the collective intentionality debate leads to the suspicion that those approaches are too cognitivist to account for the real psychological processes involved in collective action (Tollefsen and Dale, 2012). In our view, this worry can easily be overcome when combining research on collective intentionality with work from other fields, thereby also accounting for dimensions of collective action that one tends to overlook when exclusively focusing on the level of intentionality.

Second, we worry that the debate on collective intentionality tends toward a specific version of *internalism*. We use this term in a specific sense here, referring to the idea that only internal factors (such as beliefs, desires, and intentions) play a direct role in the explanation of action. External factors (such as obstacles and opportunities in the environment) are said to contribute only indirectly to the shaping of action through the modification of beliefs, desires, and intentions. By contrast, research from ecological and situationist social psychology and embodied and embedded cognitive science focuses on the role of environmental factors in shaping (and explaining) human action (Mischel, 1968; Ross and Nisbett, 1991; Doris, 2002). Our worry is that the internalism of the collective intentionality framework hinders the combination with approaches that account for environmental factors in the explanation of collective action.

We can illustrate these points by way of examples. At the Tour the France, the peloton smoothly flows through a roundabout, splitting up before and reuniting after the obstacle. In a basketball game, the entire defense rotates to help when a defender gets beat to the basket, protecting the rim and hurrying to guard open shooters. During market validation, a startup company modifies its product design to better fit with costumer preferences. In all these cases, a whole collective swiftly responds to obstacles and opportunities provided by the environment. The peloton reacts to the road furniture on its way. A basketball team responds to the moves of the opposing team. A startup responds to what market research indicates. This is also true for classic examples in the collective intentionality debate: On closer inspection, preparing a sauce hollandaise together (Searle, 1990), or even walking together (Gilbert, 1990), require constant coordination between individual human bodies who need to bring along rather sophisticated bodily habits and skills. Now, we are not claiming that the collective intentionality framework cannot account for those examples. All we want to do is to point out that an exclusive focus on intentionality in the explanation of collective action might come with problematic consequences. Let’s discuss this further by considering how proponents of the collective intentionality framework might respond to our examples. There are at least three possible lines of response.

(a) Some might argue that some of our examples are not cases of collective action after all – either because they do not involve intentions that are properly collective or because they are not instances of intentional action in the first place. However, this response seems counterintuitive, unless one shares some very specific theoretical commitments about what makes an action intentional and/or collective.

(b) Others might point out that our examples include different types of collective action that require different explanations, and that only some of them are relevant to the collective intentionality debate. However, a proponent of such a response would already admit that the collectively intentionality framework is not able to account for all of the phenomena which one would pre-theoretically call “collective actions,” and thus, that it has only a limited applicability. Moreover, we are ultimately suspicious of the claim that there are fundamentally different types of collective action that require very different and maybe even incompatible explanations. We consider it more plausible and appealing to assume that there are several explanatory mechanisms that account for various *aspects* of collective action, which are, however, all relevant for all collective actions.

(c) Finally, some might claim that all of our examples can indeed be accounted for in terms of collective intentions at the end of the day. Here, the question is if an explanation that *solely* focuses on the level of intentionality is really the best explanation. For instance, the way how the peloton reacts to obstacles on its way does not seem to be fully specified by collective intentions. Of course, it is reasonable to assume that the riders have a collective intention to avoid crashes, and the collective intentionality framework is ideally suited to account for that fact. Yet this does not specify how the peloton copes with opportunities and obstacles on its way. The rider in front just experiences affordances for the collective and reacts to them (see the section “Ecological Social Psychology” below), and others follow. The immediate action-guidance provided by the environment seems to offer a simpler and better explanation of the relevant action patterns than an exclusive reference to intentions. By contrast, exclusively appealing to collective intentions comes at the risk of overlooking the dynamical interaction of a collective with its environment. Similarly, a couple cooking a sauce hollandaise together has most likely the collective intention to do so, but how they perform this action is guided by embodied habits and routines that allow each of them do their part. The same is true for all the other examples: collective intentions certainly play a role in them, but our explanation of those collective actions becomes much stronger if we combine collective intentionality with other explanatory mechanisms.

We take this to show that collective intentions might be nothing more and nothing less than one factor among many others in the explanation of collective action. Our worry is that the exclusive focus on collective intentions tends to conceal the presence and importance of other factors, such as environmental influences, dynamical self-organization, social identities, and others. The collective intentionality framework offers a detailed analysis of one explanatory mechanism, but our explanation of collective action can become much stronger when it also includes other explanatory mechanisms.

## Team Reasoning

At this point, we move to another theory that, at first glance, appears tailor made for at least some of the examples mentioned in the previous section. In a prominent paper introducing the theory of team reasoning, Robert Sugden presented team reasoning as a solution to a situation in sports that closely resembles our basketball examples. He labeled it “the footballers’ problem” (Sugden, 2000, p. 179): imagine a scenario in a football game from the perspective of two attacking players. Player A has the ball, but a defender is putting pressure on him. Player A’s teammate, Player B, has two directions in which she could run in order to receive a pass from player A. To complete the pass, player A must pass the ball in the same direction in which player B will run. Sugden summarizes the options as follows: “Suppose that the move to the right puts B into a slightly better position. Say that the probability that the pass will result in a goal is 10 percent if both choose left and 11 percent if both choose right. If one chooses right and the other left, the probability is zero. What should each player do? The answer seems obvious: each should choose right” (Sugden, 2000, p. 179). Although this solution seems obvious to the lay person, *traditional* game theory (which Sugden sees in need of improvement) does not allow to infer this conclusion. For according to traditional game theory, each player solely reasons from her individual perspective. Under such premises, what side she should choose depends on what she expects her teammate to do; and the same goes for her teammates. And since those expectations are uncertain, they are caught in a vicious circle.

The theory of team reasoning solves this problem (Bacharach, 1999; Sugden, 2000, 2003). The main idea is the following: in cases of identification with a group, people do not reason from their individual perspectives, but rather use a distinctive mode of team reasoning. In other words, they make decisions from the perspective of the team. This contains an idea we consider very important for the explanation of collective action: *We often make sense of the world from the perspective of a collective*.

However, the model of team reasoning, when *not* combined with other approaches, has significant problems and lacunas in its explanation of collective action. As the limitations of the team reasoning approach closely resemble those of the collective intentionality framework, we only address them briefly, focusing on aspects which we have not dealt with above. Research on the function of practice in team sports shows that practice enables the players on a team to develop a repertoire of moves which they can enact as a unit. It allows all players to interpret the game in the same way and to immediately respond to evolving situations together (Brümmer, 2015; Alkemeyer and Bröckling, 2018). This indicates that sport teams primarily function through processes of dynamical self-organization based on the coordination of properly trained individual bodies. The team reasoning approach does not offer conceptual resources to account for how practice shapes team members so that their coordination runs smoothly and unambiguously. As a consequence, it tends to construct “the footballers’ problem” as if players were left in a vacuum in which they must make up their minds between seemingly arbitrary options. Thereby, it ignores the fact that individuals are in constant interaction with each other, and that they can rely on a background of common routines and habits, and that these synchronic and diachronic patterns provide them with numerous cues for coordinating their action. Thus, we are worried that the team reasoning approach, when on its own, cannot address all relevant aspects of collective action, as it lacks the conceptual tools to account for the constant self-organization of a collective and the action guidance provided by the environment. By contrast, practice theory, which we will discuss in the section “Practice Theory,” suggest that, once team members are adequately subjectivized through common training, movements of team members are smoothly coordinated by their matching interpretations of environmental cues, allowing a collective to quickly respond as a unit to challenges it faces. Thus, if two players were to face Sugden’s version of the “the footballers’ problem” in explicit terms, this would be a sign of a serious break-down in the normal functioning of a team.

In sum, the theory of team reasoning adds an important element to the explanation of collective action: individual human bodies often come to evaluate their options from the perspective of a collective and act on that basis. On its own, however, it only offers limited tools for the explanation of collective action.

## Social Identity Theory

We now turn to a third approach which is committed to cognitivism, namely the self-categorization theory of social groups, which is particularly prominent in social psychology (Turner, 1982; Turner et al., 1987). Self-categorization theory strives at a psychological understanding of social groups based on the self-identification of individuals: “It proposes that a social group can be defined as two or more individuals who share a common social identification of themselves or, which is nearly the same thing, perceive themselves to be members of the same social category” (Turner, 1982, p. 15). The gist of this approach is that when a self-categorization becomes salient for an individual, this evokes a particular social identity. When a social identity is thus activated, an individual appraises a situation not with respect to her individual concerns, but with regard to the concerns of the relevant group. Thus, social identity theory is fully compatible with the team reasoning approach and further contributes to our understanding of how individuals can come to evaluate a situation from the perspective of a collective and act on its behalf. And it adds broad empirical support to the claim that individuals regularly evaluate situations from the perspective of groups with which they identify (Smith et al., 2007; Mackie and Smith, 2017).

However, as in the cases of the frameworks of collective intentionality and team reasoning, we consider the explanatory power of social identity theory to be limited as long as it is not supplemented by other aspects. Most importantly, self-categorization theory only accounts for the intellectual aspects of group participation. It has limited resources to conceptualize the fact that belonging to a group might not be a simple matter of self-identification, but a complex and dynamic result of diverse routines, habits, affective attachments, etc. For instance, self-identifying as a proficient racing cyclist is not enough to smoothly ride as part of a peloton. If someone is not properly trained to follow a large set of implicit and explicit norms and conventions and to perform a variety of difficult techniques, his presence in the peloton will quickly lead to a crash.

Thus, we suggest reframing the notion of social identity in view of the importance of embodied interactions with the social environment. With this suggestion, we can build on an interactional approach to social identity which links social identity to dynamical system theory (Barandiaran et al., 2020). According to our proposal, developing a social identity requires participation in relevant perception-action loops in which the social identity is enacted. At least in regular cases, self-identification as member of a social group is the result of repeated social interactions, in which one enacts and thereby performatively establishes one’s membership in a group (Butler, 2015). This includes being continuously recognized as having a certain role and identity in those interactions. Such recognition depends on processes of coordination and participatory sense-making (to be explored in the section “Participatory Sense-Making”). For instance, developing the social identity of being the co-founder of a startup usually implies participating in numerous meetings in which one is addressed in that role. In short, we suggest that social identities emerge and are stabilized within relevant patterns of interaction.

At the same time, social identities can easily be activated and thereby facilitate participation in social interactions. Having the relevant social identity allows individuals to anticipate their participation in collective perception-action loops. For that reason, five proficient basketball players can quickly form a team even if they have never played together before. Thus, the key contribution of social identity theory to the explanation of collective action is that it addresses the psychological processes that enable individuals to see the environment from the perspective of the collective, even before actual coordination with others occurs. It accounts for the fact that individuals regularly understand themselves as members of a collective without having the – more sophisticated, scholarly – view that collectives are self-organizing systems that exist only in and through the dynamical processes that enact them.

## Ecological Social Psychology

We now move to a fourth approach to collective action which in many respects is the very opposite of the previous three approaches. Whereas the collective intentionality framework, the team reasoning approach, and social identity theory are all rooted in cognitivism, ecological psychology deliberately understands the mindedness of organisms in a very thin way, and deemphasizes the importance of intentions, goals, representations and mental states in general.

Ecological psychology conceives of the environment in terms of affordances. According to Gibson (2015, p. 119):

[t]he affordances of the environment are what it offers the animal, what it provides or furnishes, either for good or ill. The verb to afford is found in the dictionary, the noun affordance is not. I have made it up. I mean by it something that refers to both the environment and the animal in a way that no existing term does. It implies the complementarity of the animal and the environment.

While the exact meaning of “affordance” is controversial among ecological psychologists, it is uncontroversial that the concept characterizes the environment in terms of possibilities for action that exist in relation to an organism. Depending on the bodily constitution and abilities of an organism – its so called “effectives” (Turvey and Shaw, 1979) –, certain features in its surroundings become affordances for the organism. For example, a mouse affords chasing and eating to a cat, but not to a frog. A tree affords climbing to a squirrel, but not to a dog. According to ecological psychology, explaining the perception and action of an organism requires investigating the environment it is situated in, and not (primarily) its intentions, goals or other inner states.

Ecological psychology is originally a theory of individual perception, and not of collective action. However, ecological psychology has also been applied to the topic of action. It has been suggested to develop a sensorimotor approach to behavior, according to which action consists in constant loop of perceiving (multiple) affordances, reacting to them, perceiving new affordances, and so on (Weichold, 2018). To explain highly interactive actions such as Aikdo, this process has to be thought of as radically dynamical, where new affordances emerge continuously during the interaction (Kimmel and Rogler, 2018). And to be clear, ecological psychologists hold that “actions are not caused by the environment or elicited by stimuli, but are the animals’ means to utilize the affordances in their environment” (Withagen et al., 2012, p. 252).

Moreover, ecological psychology has also been developed further to explain social phenomena, and this is why this approach is of importance for this paper. Already Gibson (2015, p. 120) wrote that “[t]he other animals afford, above all, a rich and complex set of interactions, sexual, predatory, nurturing, fighting, playing, cooperating, and communicating.” To conceptualize these kinds of affordances which invite social behavior, Valenti and Gold (1991) have coined the term “social affordances.” A related class of affordances are so-called “joint affordances,” which are affordances that require more than one individual to be realized (Knoblich et al., 2011, who summarize the work of others). An example of a joint affordance is two-handled saw, which can only be used by two persons working in tandem.

We consider these conceptions from ecological psychology as crucial for an investigation of collective action. For, they help seeing the environment in the right way, so that it becomes clear that looking at the environment is essential if one wants to explain collective action successfully. Collective action seems to depend crucially on what the environment affords socially, a fact that is overlooked by the approaches discussed so far. However, we do not think that ecological psychology – as sketched this far – is already able to offer a successful explanation of collective action. Our main concern is that ecological psychology (again, as sketched this far) is overly individualistic. It is only individuals who interact with others, and only individuals who need other individuals to perform tasks together. What is missing is the idea that a whole collective can be in action, and that there are affordances not only for individuals, but for a collective as a whole.

Fortunately, some ecological psychologists have started accounting for this lacuna. To this end, ecological psychology has been combined with dynamical systems theory (Vallacher et al., 2002; Riley et al., 2011). The idea is to appeal to dynamical self-organization (Dale et al., 2014). Different organisms can be coupled together in a specific, structured, dynamical way so that they form a unit which is regulated by the behavior of the involved organisms and, at the same time, regulates their behavior. An example is a sports team. The behavior of the players is coupled together so that they form a team and play attack and defense moves in a structured way. The players are constantly reacting to each other and compensate for each other’s errors. Thus, the behavior of the team is regulated by the behaviors of the individual players, but the fact that they are part of team is what regulates the behaviors of the players in the first place. The behavior of each player is constantly influenced by and makes only sense because of her being part of the team. Ecological psychologists call these self-organizing systems “interpersonal synergies” (Bernstein, 1967). A synergy is “a task-specific organization of elements such that the degrees of freedom of each component are coupled, enabling the degrees of freedom to regulate each other” (Araújo et al., 2016, p. 167). Importantly, a consequence is that there can be affordances the subject of which is not an individual, but a synergy. These affordances can be called “collective affordances” (Leonardi, 2013; Weichold and Thonhauser, 2020). Lifting a piano, for example, is not an affordance for a lone individual, but it is an affordance for a group of furniture movers.

This combination of ecological psychology and dynamical systems theory is highly relevant for the development of a promising account of collective action, as it contributes important explanatory resources to the investigation of collective action. First, it contributes its insight that the environment is action guiding. Second, it contributes its conceptualization of the environment in terms of social, common, joint and collective affordances. And third, it contributes the appeal to interpersonal synergies and dynamical self-organization in order to rethink the nature of the subject of collective action.

However, problems and lacunas remain even in this augmented version of ecological social psychology, which is why it is necessary to synthesize ecological social psychology with other approaches to collective action. The key problem is that ecological social psychology overreacts in its opposition to cognitivism and thus throws out the baby with the bathwater. We agree that it is important to not *overintellectualize* the mind in (collective) action. However, ecological psychology is at least in danger of *underintellectualizing* the mind in (collective) action. After all, collective intentions and goals are important to explain the macro structure of (most) collective action, but it is at least controversial if and how they could be integrated into social ecological psychology. Playing a sport like football – the microstructure of which can indeed be explained well by appeal to ecological social psychology (Davis, 2016) – makes only sense against the background of common goals like winning the game. Moreover, analyzing the minds of the members of a synergy can be crucial for explaining how synergies emerge and continue to exist. For instance, it is important that the members have certain roles on the team, experience themselves as part of the team, and see the world from the perspective of the team (Weichold and Thonhauser, 2020). And this, in turn, might become intelligible if one looks diachronically at the history of the formation of the team. These are crucial points ecological social psychology has so far not accounted for. Thus, ecological social psychology on its own cannot offer a convincing explanation of collective action. But it is indispensable to include its insight into a successful explanation.

## Participatory Sense-Making

The conception of participatory sense-making, which we address next, was originally not intended as a contribution to the explanation of collective action. Rather, it was introduced as an enactivist approach to social cognition (De Jaegher and Di Paolo, 2007; De Jaegher, 2009; McGann and De Jaegher, 2009; De Jaegher et al., 2010). However, it develops a claim that is more broadly applicable, and we suggest adopting this claim to fill an important lacuna left by all approaches to collective action discussed so far. The approaches discussed so far do not provide conceptual resources to address the issue of how a collective perspective is constituted in the first place. When a collective and all its members view the world from the perspective of a collective and perceive collective affordances, how does this collective perspective come about? This is where the participatory sense-making approach comes into play. An augmented version of participatory sense-making will help us explain how the *perspective of a collective is dynamically constituted through processes of collective sense-making*.

Participatory sense-making is an adoption of the enactivist notion of *sense-making* to the social domain (De Jaegher and Di Paolo, 2007). According to enactivism, all organisms should be understood as sense-making systems. That means that all organisms bring about their environment as meaningful; they perceive objects in their environment as more or less relevant, as more or less mattering to them. In its most primitive form, the meaningfulness of the environment correlates with the capabilities and needs of an organism. The environment of a tick, for instance, contains only few meaningful elements which are all centered around enabling it to find a suitable host for sucking blood (von Uexküll, 1926, 2010). Thus, even the simplest of animals have a sense of what is mattering to them, of what is relevant to their well-being (Colombetti, 2014). This may be simple things like receiving nutrition from blood-feeding, as in the case of the tick, or much more complex issues as one would expect in humans and other higher animals. For instance, this research program has recently been extended to account for linguistic activities (Di Paolo et al., 2018).

Building on this notion of sense-making, the question arises how the sense-making of individual organisms is modified when they engage in social interaction. For this transfer of sense-making to the social domain, De Jaegher and Di Paolo (2007, p. 490) adopted elements of dynamical systems theory, which has already been discussed in the previous section in the context of ecological social psychology. This is a fortunate coincidence for our endeavor, for the shared background in dynamical systems theory allows for seeing compatibilities between this branch of enactivist cognitive science and ecological social psychology. De Jaegher and Di Paolo’s main import from dynamical systems theory is a dynamical understanding of the coordination between coupled systems. They define coordination as “the non-accidental correlation between the behaviors of two or more systems that are in sustained coupling, or have been coupled in the past, or have been coupled to another, common, system” (De Jaegher and Di Paolo, 2007, p. 490). The strength of dynamical systems theory is its broad applicability: coordination is a very basic mechanism that is omnipresent in physical and biological systems. A famous example in the physical domain is the synchronization of two pendula when coupled by a suitable medium. Even in the case of higher animals, coordination between coupled systems requires little cognitive skills, often takes place without awareness, and can hardly be avoided. This is evidenced by findings showing that humans come to unconsciously align their mimic, gestures, and bodily postures when coupled to each other (Chartrand and Bargh, 1999; Hess and Blairy, 2001).

While this dynamical concept of coordination is able to explain simple processes of behavioral alignment, it has originally not been able to account for more demanding forms collective action. That is why De Jaegher and Di Paolo (2007, p. 493) suggest moving from the basic level of coordination to the domain of proper social interaction. What distinguishes social interaction from other coordination processes is that in social interaction, previous coordination patterns influence the disposition of interactors for future coordination processes. A past coupling of a pendulum does not alter its mechanism, and thus, it does not affect the pendulum’s future synchronization patterns. By contrast, earlier social interactions influence later social interactions. For instance, the coupling of two experienced dancers is very different from a coupling involving a novice. “This is due to the fact that the interactors are highly plastic systems that are susceptible to being affected by the history of coordination. When this double influence is in place (from the coordination onto the unfolding of the encounter and from the dynamics of the encounter onto the likelihood to coordinate) we say we are in the presence of a social interaction” (De Jaegher and Di Paolo, 2007, p. 492). *Social interaction* is an emergent and self-organizing process of coordination between two self-organizing interactors, in which they “organize themselves according to the two avenues of influence just described: the agents sustain the encounter, and the encounter itself influences the agents and invests them with the role of interactors. […] It constitutes a level of analysis not reducible, in general, to individual behaviors” (De Jaegher and Di Paolo, 2007, p. 492). Thus, social interaction is both structured by the interactors’ dispositions and structures those dispositions.

Now, importantly for our context, social interaction also influences the sense-making of interactors. To begin with, their individual sense-making becomes coordinated in social interaction. Through processes of social interaction individuals come to see similar elements of the environment as meaningful and relevant. In other words, social interaction leads to coherence between the sense-making activities of involved individuals. This coherence of sense-making activities, in turn, opens-up new domains of social sense-making that are not available to un-coordinated individuals. De Jaegher and Di Paolo (2007, p. 497) use “participatory sense-making” to refer to the coordination of sense-making activities that not only influences individual sense-making but also leads to the possible emergence of new domains of sense-making that only emerge once a threshold of coherence between individual sense-making activities is reached. Partner dance, for instance, requires that both partners interpret the cues of each other and the surrounding in a highly synchronized way which is only possible based on a high degree of coherence of their sense-making processes (Kimmel, 2012, 2015).

The participatory sense-making approach focuses on the social interaction of dyads and does not yet address the issue of collective action. However, we suggest taking this proposal all the way up to the collective level. To be sure, some researchers in the field might not be entirely happy with this (Stapleton and Froese, 2015), but we propose that it will lead to a convincing conception which one can henceforth work with. After all, all the necessary tools are already there: As Fuchs and De Jaegher (2009) have argued, social interaction can result in the coherence of sense-making activities becoming so high and the corresponding coordination patterns so tense that a new sense-making unit emerges, the social interaction as a whole. In such instances, in which the social interaction becomes a new “center of gravity” (Fuchs and De Jaegher, 2009, p. 476), we suggest speaking of *collective sense-making*. The strength of the enactive approach is that it allows to zoom in on the ongoing social interaction between individual human bodies, processes which dynamically enable and maintain collective sense-making activities. Moreover, when combined with key insights from dynamical systems theory, the enactive approach is well-equipped to consider the top-down and bottom-up restraints structuring collective sense-making.

On the other hand, its explanatory resources are rather thin when it comes to accounting in detail for the diachronic dimension of how human bodies are modified to make them suitable candidates for social interaction in the first place. Indeed, Steiner and Stewart (2009) have argued that the participatory sense-making approach is problematic because it cannot account for the fact that social interactions do not just happen when two human beings interact. Instead, what is crucial about human social interactions is that they are social in the deeper sense that they are always already structured and even constituted by social rules that exist prior to a particular interaction. A dance, for instance, can only take place in a social practice in which the social institution of dancing and the social roles of dancers exist. While we agree with Steiner and Steward on this topic, this is at the same time the domain in which the final approach, which we want to discuss, has its core strengths. And to be sure, this final approach can also be conceived of as being very well compatible with the enactive approach. As Di Paolo and De Jaegher (2017) have emphasized, participatory sense-making is meant to take into account such a diachronic, larger-scale shaping of particular social interactions.

## Practice Theory

Like the previous two approaches, practice theory is originally not a theory of collective action, but an innovative account of sociality. However, it entails explanatory mechanisms that should, according to our judgment, be included in a successful account of collective action. Moreover, practice theory has recently been applied to the topic of collective action, so that there is, by now, a practice-theoretical research program on collective action.

The term ‘practice theory’ has been invented as an umbrella term to summarize common thoughts and methods from a variety of thinkers such as Wittgenstein, Heidegger, Bourdieu, Giddens, Butler, and Schatzki (Reckwitz, 2003). The approach is less prevalent in philosophy, but common in social theory, cultural theory, and ethnography, and it is so successful that a “practice turn” has been proclaimed (Schatzki et al., 2001). The key concept is that of social practice. A practice is, according to a famous characterization, a “temporally unfolding and spatially dispersed nexus of doings and sayings” (Schatzki, 1996, p. 89). It consists of repeated performances which are guided by implicit norms and bodily know-how and which take place in socio-material environments (Reckwitz, 2003). A practice is seen as more fundamental than individuals and ‘the society’. Indeed, practice theory holds that individual human beings only become the subjects they are in and through social practices, and that ‘the society’ is nothing but a configuration of social practices. Practice theory thus explains actions in a way that is fundamental different from the way of other accounts. It does not put emphasis on the intentions of the involved agents (even though it does not deny that they might play a role). Rather, what practice theory considers crucial is that an action re-enacts a familiar performance (e.g., raising one’s hand in a discussion to make a point), that an acting human being is equipped with the right bodily know-how (e.g., how to raise one’s hand not aggressively, but politely), that the action takes place in the right socio-material environment (e.g., in a classroom), and that the acting human being is recognized and treated by others as an agent (e.g., respected as a student worthy of making a point in a discussion).

While this account is already helpful for the understanding of (collective) action, we want to call attention to one specific explanatory mechanism that will be of special importance for our synthetical account of collective action. It is the explanatory mechanism of *subjectification* (Foucault, 1977; Butler, 1997). The idea is that human beings are first and foremost not intentional agents or responsible subjects, but ‘living bodies’ or ‘brain-body-systems.’ Such a brain-body-system can *become a subject* through a process of subjectification. To make this happen, the brain-body-system has to be subjected to the norms of a practice, habitualize following them and acquire the respective bodily know-how. Then, others (with the right positions of power in the respective social practice) can see and treat the respective brain-body-system as a subject. For example, a student of law has to subject her whole behavior and way of thinking to the social norms of her profession in order to become, at one point in time, recognizable as a professional lawyer. This idea will be important for our synthetical account of collective action because it can explain how particular human beings can develop their social identities in such a way that they become suitable interactors in social interactions that can lead to the emergence of collective sense-making: Their respective social identities are the results of a diachronic and always on-going process of subjectification.

Some practice theorists have recently applied the idea of subjectification to collectives, thus starting the mentioned practice-theoretical research program on collective action (Marschelke, 2019; and all of the contributions in Alkemeyer et al., 2018). The central thought is that not only individual subjects, but also collectives come into being through a process of subjectification (Alkemeyer and Bröckling, 2018). To distinguish the formative processes shaping a collective from individual subjectification, those dynamics on the level of the collective can be called *collectification* (Marschelke, 2019). Collectives are seen here not as static entities, but as emergent systems that come into being in virtue of dynamical processes – a view which practice theory shares with dynamical systems theory. Thus, practice theory directs us to investigate the social processes that bring about and sustain a collective. It is crucial that interacting brain-body-systems are *recognized* as a collective, both by the members of the collective and by other individuals and collectives. This recognition is a permanently on-going process of iterated performances. For instance, in the case of the collective of a family, the stabilizing, re-enacting performances occur constantly in practices such a having dinner together or celebrating birthdays with the family (Grundmann and Wernberger, 2015). Additional roles in collectification are played by artifacts (e.g., family photos) and the socio-material environment in general (e.g., sharing a flat). Finally, a particularly important feature that helps to enact the collective as a process is the symbolic fiction that the collective is a fixed entity (Alkemeyer and Bröckling, 2018). In order to enact a collective, the members of the collective-to-be often have to believe that there already exists the collective of which they are members. Thus, by (falsely) believing in the existence of a collective as a fixed entity, its members-to-be and others contribute to actually bringing about a collective as a self-organizing system that exists in dynamical processes.

The application of practice theory to collectivity is a rather young research program, and so it is no wonder that there are many lacunas. Practice theory lacks an elaborated understanding of how to think of a collective as a self-organizing system – something that has already been provided by adaptations of dynamical systems theory in ecological social psychology and in enactivist cognitive science. Practice theory also lacks the conceptual resources to explain how the members of a collective make sense of their environment together – something which is explained by the enactivist approach as well. Likewise, practice theory is in danger of neglecting that collectives and its members experience their environment from the perspective of the collective – something which has been elucidated by social identity theory and the theory of team reasoning. Practice theory does not account in detail for how the environment guides collective action – something which is clarified by ecological social psychology. Practice theory has moreover not yet analyzed thoroughly enough the mental and social constitution of the members of a collective – something which has been done better by social identity theory and the collective intentionality framework. All this provides further evidence that a successful explanation of collective action requires a synthesis of existing research. The practice theoretical approach to collectivity is fruitful, but there would be a much stronger approach if the two mentioned explanatory tools from practice theory were combined with the successful resources from other research programs.

## A Synthesis

In this final section, we propose a synthesis of the mechanisms for explaining collective action which have been extracted from the different research programs, yielding, to our mind, a first proposal of how core ideas from the different research programs can be fruitfully synthesized. We suggest that the resulting synthesized framework has more explanatory power than each of the previously discussed research programs on its own. We therefore invite researchers to also work toward such a synthesized framework, or to at least appreciate how their favorite theoretical approach to collective action can benefit from being integrated with resources extracted from other approaches. To be sure, we understand our synthesis as a first step toward a new integration, and not as the last word on the topic. As a matter of presentation, we will dissect our proposal into its components, the in total eight explanatory mechanisms, and present them one after the other. It should be noted, however, that we take all these explanatory mechanisms to be interconnected with each other and to provide, if taken together, one grand explanation of collective action.

To avoid any confusion about *collectives* as the agents of collective actions, we remind the reader that collectives might have different shapes and forms. To begin with, they can vary in size, from a couple preparing a meal to a large enterprise developing a new product. A collective’s organization might also come in different degrees, from loose and spontaneous coordination, as in the case of strangers pushing a car out of a roadside ditch, to rigid organization like in a military unit. Relatedly, the organization might involve flat hierarchies or a strict chain of command. Moreover, the inner and outer interactivity of collectives can be on different scales, from sport teams needing to react to emerging situations and to coordinate with each other in split seconds, to work groups which mainly communicate via e-mail. There are also differences in how cognitively demanding it is to participate in a collective. Similarly, the degree of required bodily skill and inter-bodily coordination might vary. All of this implies that collective action is not an on/off-question, but rather a gradual matter, depending on the degree of self-organization of a collective.^1^ Furthermore, a collective can have other collectives as its parts, sometimes in a more cooperative, sometimes in a more competitive relation to each other. For instance, a startup usually consists of smaller working units aiding each other, while the peloton at the Tour de France consists of teams which share the goal of avoiding accidents but compete for winning the race. We do not claim this list to be exhaustive. It is just meant to give an idea of how broadly applicable we consider our concept of collective to be.

We propose to take seriously the intuition that *collective actions are genuinely performed by collectives*. While some researchers, especially within the collective intentionality debate, deserve praise for having preserved this intuition in their theories (Gilbert, 2009; Schmid, 2009; List and Pettit, 2011), most thinkers fear that such a position would imply the postulation of mysterious collective entities. To them, our approach might be of special interest. For our approach shows a (new) way of how to follow the intuition that collective actions are performed by collectives, without thereby postulating mysterious entities. To show this, we refer to dynamical systems theory. According to our definition, *collectives* are *emergent self-organizing systems*. They are dynamically constituted, ecologically situated, and consist of two or more appropriately subjectivized brain-body-systems. As explained in the discussions of ecological social psychology and participatory sense-making, we adopt *dynamical self-organization* (Vallacher et al., 2002; Dale et al., 2014) (explanatory mechanism 1).

Dynamical self-organization implies bottom-up and top-down restraints. As a collective is dynamically constituted by the interactions of individual brain-body-systems, the system’s complexity stems from interaction patterns which depend on the action capabilities and interaction disposition of its members. Thus, the constitution of its members has bottom–up restraints on how a collective is organized and what it can do. For instance, the ability of a startup to develop a new software, and how it will go about this task, will crucially depend on its employees’ coding skills as well as their collaboration skills. Similarly, if and how a couple is capable of preparing a sauce hollandaise depends on, among other things, its members proficiency in whisking egg whites. However, the emerging self-organization “may arise in a fashion that cannot be predicted solely from knowledge of the individual elements in isolation” (Vallacher et al., 2002, p. 266). Since the dynamical interactions in a self-organizing system are non-linear in nature, the action capabilities of a collective are different from the sum of the action capabilities of its members. Effective communication patterns and a motivating team culture might make a startup more capable than one would expect when considering only the skills of its employees, while the emergence of sluggish procedures might lead another startup to underachieve despite highly skilled members. Moreover, the organization of a collective has further top–down effects on its members. An organizational structure that facilitates mutual learning will improve the problem-solving skills of team members, just as an open team culture will improve the communication patterns within the team. Thus, the dynamical self-organization of a collective not only depends on the action capabilities and interaction patterns of its members, it also shapes and transforms those dispositions. In sum, dynamical self-organization allows explaining how the agent of a collective action, a collective, comes about.

Yet to fully comprehend how a collective as the agent of collective actions comes about, it is crucial to supplement this general idea of dynamical self-organization with other explanatory resources. One important aspect is accounting for the emergence of collectives in social space. Recent research within practice theory shows that social processes of *recognition* play an important role in bringing about and sustaining a collective (Alkemeyer et al., 2018; Marschelke, 2019). How an interconnected bunch of brain-body-systems is seen, both by the involved brain-body-systems and by others, has an effect on the constitution of a collective. For a startup to emerge, it is usually the case that co-founders already envision themselves as a successful collective, before the actual collective has emerged through sustained internal and external dynamics of interaction. Moreover, a startup can only be brought about and sustained when it has the right socio-material environment and the ongoing recognition and support of others (investors, advisors, costumers). It is through such ongoing internal and external interactions that the dynamic shaping of a collective takes place. This explanatory mechanism can be called *collectification* (explanatory mechanism 2).

To repeat our central point, understanding collectives as dynamically constituted self-organizing systems allows to account for the intuition that *the agent of a collective action is, in fact, the collective.* The emergence of a collective implies the emergence of new action capabilities and action opportunities that do not exist for individuals. In other words, there are affordances the subject of which is not an individual, but a collective as a whole. Those affordances can be called “*collective affordances”* (Leonardi, 2013; Weichold and Thonhauser, 2020) (explanatory mechanism 3). This concept of collective affordance allows to account for the ecological situatedness of collective action. In collective action, it is the collective as a whole that interacts with its environment. Now, affordances are relative to the “effectives” (Turvey and Shaw, 1979) of a subject, and in the case of collective action, that subject is the collective. Collective affordances are ontologically depended on collectives, i.e., they only emerge relative to the effectivities of collectives. One can thus explain collective action by referring to the interaction of a collective with the collective affordances provided by its environment. For instance, during a bicycle race, the slipstream available within a sufficiently large group will make an escape attempt appear worthwhile or not. Narrow streets afford a large peloton to slow down, while they afford a small escape group to drive through at full speed. Collective affordances dynamically emerge and vanish as a collective interacts with its environment.

However, why does a collective perceive and react to collective affordances? We suggest that collective affordances depend on a *collective perspective* (explanatory mechanism 4), i.e., on how a collective perceives its environment and enacts the action opportunities which it provides. And again, what a collective can do depends on the specific action capabilities which the collective takes itself as possessing. These assumed action capabilities of a collective are different from the action capabilities of its individual members. Depending on its size and proficiency, for instance, a startup will see different business opportunities. Similarly, preparing a multi-course meal for a full restaurant will only be an affordance for a sufficiently large group of cooks.

But how is a collective perspective constituted in the first place? Here, enactivist cognitive science allows us to zoom into the processes of *collective sense-making* (explanatory mechanism 5) through which a collective perspective is dynamically constituted. The coupling of participants during a social interaction, taken together with other processes such as collectification, can gain so much momentum that the social interaction as a whole acquire macro-level properties that make it a new agential center. Of course, the discussed processes are interrelated: It is the ongoing enactment of a collective perspective via collective sense-making that allows collective affordances to show themselves.

In collective sense-making dynamics, *collective goals and collective intentions* often play a role (explanatory mechanism 6). They can be a crucial part of the inner dynamic of a collective, and thus part of a collective’s orientation within its environment. For instance, a startup might have the collective intention of launching its new website next week. Cyclist in an escape group might share the collective goal of outpacing the peloton. A couple might prepare a sauce hollandaise because they have the joint intention of enjoying the evening together.

So far, we have explained collective action by explaining how a collective interacts with its environment, and by explaining how a collective emerges in the first place. However, we have not yet said much about the individual members of a collective. But of course, the emergence and continuity of a collective requires that there are at least two appropriately subjectivized brain-body-systems that interact in relevant ways and that identify themselves as parts of the collective. However, identification with a collective can come in various degrees, depending on the type of interaction and an individual’s role within a team. As Pacherie (2011) has argued, there might be collective actions where the involved individuals lose a sense of individual agency and just feel a sense of collective agency. This would be a full identification and might yield a dense self-organization of the emerging collective. However, Pacherie points out there might also be many other cases. For example, a member of a collective who acts within her role might still have a high sense of individual agency and a very low sense of collective agency – which is, according to Pacherie, particularly true of agents with a very low status in very hierarchical groups. Following an augmented version of social identity theory, one can say that a proper identification with a collective requires, as a precondition, that individuals have obtained role-specific bodily know-how and interaction dispositions, something that might be called *embodied social identities* (Weichold and Thonhauser, 2020) (explanatory mechanism 7). This non-explicit, bodily readiness to play one’s part is the identification that is needed for an individual to be part of a collective. As embodied social identities, action patterns can readily be reactivated and enable individuals to smoothly contribute to collective action. The explanatory mechanism of embodied social identities can thus explain why an individual brain-body-system is ready to switch from individual routines into viewing the world from the perspective of a collective and doing her share in the collective’s endeavor. For instance, five high-level basketball players can rely on each other’s embodied social identities to immediately function as a team. Similarly, experienced dancers will quickly feel out each other rhythms and behavioral cues and soon be able to perform an advanced dance together (Kimmel, 2012). Against this background, the individual participants can see the world in terms of “embodied social identity affordances” to do their share in a collective action.

As these examples show, embodied social identities are the result of a long history of participation in relevant interactions. A brain-body-system needs to have undergone a process of *subjectification* to become a suitable interaction partner that can play her respective role within a collective (explanatory mechanism 8). Subjectification allows us to explain, via reference to a history of self-formation, how it is possible for the members of a collective to successfully play their roles within the larger unit. For instance, a basketball player, after years of training, has embodied the routines of how to perform particular movement patterns relevant for the position she is playing. When the movement patterns of the five players on the court match, they can smoothly operate as a team. Similarly, musicians train for a long time to acquire the relevant know-how to perform their part within an orchestra, and again, matching histories of subjectification enable them to play as a unit. By contrast, a social interaction will not run smoothly when participants do not possess the relevant bodily know-how and interaction dispositions. For that reason, a couple with little experience in the kitchen will likely not succeed at its first attempt of a sauce hollandaise.

This is also the place where further research on collective action from other fields might eventually be integrated into our synthesis. For instance, research from social neuroscience can explain the brain processes that are involved in collective action (Todorov et al., 2011). However, we insist that an explanation of collective action in terms of brain processes alone has to remain unconvincing, as all the other mentioned explanatory mechanisms are always relevant as well.

**FIGURE 1:**
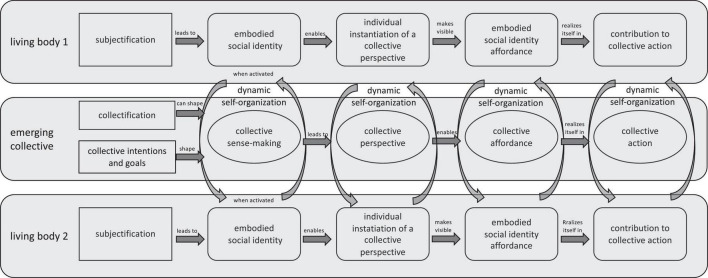
Visual illustration of the proposed synthesis.

The following figure (Figure 1) is an attempt to visually represent our proposal for an integrated account. To keep things as simple as possible, we only included two brain-body-systems. However, we hold that the same explanatory mechanisms can be found in collectives of all sizes, from dyads, as in our illustration, to large gatherings.

The explanatory mechanisms are always interacting with each other, and they are all, in their dynamical interaction, relevant for explaining collective action. But of course, within different contexts of thinking about collectives, one might have different explanatory needs. In some contexts, one might, for instance, be mainly interested in the goals of a collective. In other contexts, one might be primarily interested in the bodily interaction of a collective with its environment. However, as we hope to have made clear, to really understand what is going on in collective actions requires, in all cases, reference to the entire picture. This can be seen by thinking about the many ways in which a potential collective action can fail. Imagine a team at a cycling race. The team might be unsuccessful because they do not have the proper collective intention of working together – each team member might instead be only focused on his individual career. But even if the team has a proper collective intention, it does not automatically mean that it will be successful. This depends also on how well the team members actually work together, on their dynamical self-organization. Can they make sense together and establish a collective perspective? How well they are able to do this depends, in turn, on subjectification and collectification. Yet even if all the team members completely identify with the team and interact with each other well, a team might still be unsuccessful if it does not have a proper strategy in terms of embodied social identities, that is, in this case, if the team members do not have clear, well-trained roles that imply clear tasks of the individual team members. However, even if the inner organization of the team is perfect, it might still be unsuccessful because it does not properly interact with potential collective affordances. Maybe several important team members attempt a escape from the peloton at a route where this is hopeless, or the team members fail to see a rather promising opportunity for an escape. In sum, properly understanding collective action requires appreciating all the different explanatory mechanisms and their interactions.

## Conclusion

In this paper, we discussed some of the to our mind most relevant approaches to collective action in current debates. Of course, we were not able to discuss any single of these frameworks in all details. Rather, our goal was to provide researchers in different areas with an overview of the different approaches to researching collective action, showing what their respective strengths and limitations are, and suggesting a way how promising explanatory mechanisms can be extracted from those approaches and be integrated into a new, synthetical framework for investigating collective action. As discussed in the introduction, the real progress in recent research on collective action lies in the fact that there is, by now, a multitude of successful research programs.

Our own synthetical approach to collective action is a first proposal of how to combine the different explanatory mechanisms into an integrated account. We consider it a strength of our proposal that it is able to zoom into the ongoing processes of dynamical self-organization. But our model also allows integrating a diachronic perspective that accounts for how individuals are shaped to be suitable participants in collective actions. Moreover, our account also allows for focusing on how collectives as a whole are shaped.

However, our proposal is only a first step and much more work is needed to fully integrate the resources offered by the different approaches. To conclude this paper, let us indicate a few of the many questions that need to be addressed by future research. First, we suggest that there should be in-depth studies of how social interaction leads to the emergence of collective sense-making. What is the exact threshold at which interactive sense-making processes lead to the emergence of a new agential center? Second, we consider it crucial to further explore the role of individual and collective goals and intentions in processes of dynamical self-organization. We consider this the crucial step in synthesizing approaches that solely focus on intentionality and approaches that solely focus on sensorimotor coordination. Third, we suggest more research on how the explanatory mechanisms of subjectification and collectification shape sense-making processes. For that task, it appears most promising to combine research from enactive cognitive science and practice theory.

In sum, we hope that this paper will stimulate more transdisciplinary work on collective action. After all, we are convinced: The next step in researching collective action is to approach collectivity collectively.

## Data Availability Statement

The original contributions presented in the study are included in the article/supplementary material, further inquiries can be directed to the corresponding author/s.

## Author Contributions

All authors listed have made equal, substantial and direct, contributions to the work, and approved it for publication.

## Conflict of Interest

The authors declare that the research was conducted in the absence of any commercial or financial relationships that could be construed as a potential conflict of interest.

## Publisher’s Note

All claims expressed in this article are solely those of the authors and do not necessarily represent those of their affiliated organizations, or those of the publisher, the editors and the reviewers. Any product that may be evaluated in this article, or claim that may be made by its manufacturer, is not guaranteed or endorsed by the publisher.

## Footnotes

1 This is similar to how Korsgaard (2009) analyzes individual action: The more self-organized and integrated an individual agent is at a particular moment, the more are her doings her actions.
